# Multivariate logistic regression analysis of risk factors for birth defects: a study from population-based surveillance data

**DOI:** 10.1186/s12889-024-18420-1

**Published:** 2024-04-15

**Authors:** Xu Zhou, Jian He, Aihua Wang, Xinjun Hua, Ting Li, Chuqiang Shu, Junqun Fang

**Affiliations:** Hunan Provincial Maternal and Child Health Care Hospital, Changsha, Hunan Province 410000 China

**Keywords:** Congenital abnormalities, Prevalence, Risk factors

## Abstract

**Objective:**

To explore risk factors for birth defects (including a broad range of specific defects).

**Methods:**

Data were derived from the Population-based Birth Defects Surveillance System in Hunan Province, China, 2014–2020. The surveillance population included all live births, stillbirths, infant deaths, and legal termination of pregnancy between 28 weeks gestation and 42 days postpartum. The prevalence of birth defects (number of birth defects per 1000 infants) and its 95% confidence interval (CI) were calculated. Multivariate logistic regression analysis (method: Forward, Wald, α = 0.05) and adjusted odds ratios (ORs) were used to identify risk factors for birth defects. We used the presence or absence of birth defects (or specific defects) as the dependent variable, and eight variables (sex, residence, number of births, paternal age, maternal age, number of pregnancies, parity, and maternal household registration) were entered as independent variables in multivariate logistic regression analysis.

**Results:**

Our study included 143,118 infants, and 2984 birth defects were identified, with a prevalence of 20.85% (95%CI: 20.10–21.60). Multivariate logistic regression analyses showed that seven variables (except for parity) were associated with birth defects (or specific defects). There were five factors associated with the overall birth defects. The risk factors included males (OR = 1.49, 95%CI: 1.39–1.61), multiple births (OR = 1.44, 95%CI: 1.18–1.76), paternal age < 20 (OR = 2.20, 95%CI: 1.19–4.09) or 20–24 (OR = 1.66, 95%CI: 1.42–1.94), maternal age 30–34 (OR = 1.16, 95%CI: 1.04–1.29) or > = 35 (OR = 1.56, 95%CI: 1.33–1.81), and maternal non-local household registration (OR = 2.96, 95%CI: 2.39–3.67). Some factors were associated with the specific defects. Males were risk factors for congenital metabolic disorders (OR = 3.86, 95%CI: 3.15–4.72), congenital limb defects (OR = 1.34, 95%CI: 1.14–1.58), and congenital kidney and urinary defects (OR = 2.35, 95%CI: 1.65–3.34). Rural areas were risk factors for congenital metabolic disorders (OR = 1.21, 95%CI: 1.01–1.44). Multiple births were risk factors for congenital heart defects (OR = 2.09, 95%CI: 1.55–2.82), congenital kidney and urinary defects (OR = 2.14, 95%CI: 1.05–4.37), and cleft lip and/or palate (OR = 2.85, 95%CI: 1.32–6.15). Paternal age < 20 was the risk factor for congenital limb defects (OR = 3.27, 95%CI: 1.10–9.71), 20–24 was the risk factor for congenital heart defects (OR = 1.64, 95%CI: 1.24–2.17), congenital metabolic disorders (OR = 1.56, 95%CI: 1.11–2.21), congenital limb defects (OR = 1.61, 95%CI: 1.14–2.29), and congenital ear defects (OR = 2.13, 95%CI: 1.17–3.89). Maternal age < 20 was the risk factor for cleft lip and/or palate (OR = 3.14, 95%CI: 1.24–7.95), 30–34 was the risk factor for congenital limb defects (OR = 1.37, 95%CI: 1.09–1.73), >=35 was the risk factor for congenital heart defects (OR = 1.51, 95%CI: 1.14–1.99), congenital limb defects (OR = 1.98, 95%CI: 1.41–2.78), and congenital ear defects (OR = 1.82, 95%CI: 1.06–3.10). Number of pregnancies = 2 was the risk factor for congenital nervous system defects (OR = 2.27, 95%CI: 1.19–4.32), >=4 was the risk factor for chromosomal abnormalities (OR = 2.03, 95%CI: 1.06–3.88) and congenital nervous system defects (OR = 3.03, 95%CI: 1.23–7.47). Maternal non-local household registration was the risk factor for congenital heart defects (OR = 3.57, 95%CI: 2.54–5.03), congenital metabolic disorders (OR = 1.89, 95%CI: 1.06–3.37), congenital limb defects (OR = 2.94, 95%CI: 1.86–4.66), and congenital ear defects (OR = 3.26, 95%CI: 1.60–6.65).

**Conclusion:**

In summary, several risk factors were associated with birth defects (including a broad range of specific defects). One risk factor may be associated with several defects, and one defect may be associated with several risk factors. Future studies should examine the mechanisms. Our findings have significant public health implications as some factors are modifiable or avoidable, such as promoting childbirths at the appropriate age, improving the medical and socio-economic conditions of non-local household registration residents, and devoting more resources to some specific defects in high-risk groups, which may help reducing birth defects in China.

## Introduction

Birth defects are structural or functional anomalies at or before birth [1]. The accepted prevalence of birth defects is 2–3% worldwide [2]. The prevalence of birth defects is estimated to be 4-6% in China [3]. Birth defects have been a significant problem for health care in terms of the resources they require because of their longer life expectancy [2]. Severe birth defects significantly increase the risk of stillbirths or child deaths [4–6]. In developed countries such as Europe and the US, birth defects have been the leading cause of perinatal death and infant death for a long time [7]. WHO estimated that about 12.6% of neonatal deaths worldwide each year are related to birth defects [8]. Therefore, the study on birth defects is significant and deserves more attention.

Currently, the cause of many birth defects is still unknown, and many researchers believe that birth defects may result from hereditary polygenic defects or a gene-environment interaction [9, 10]. Risk factors for birth defects may change over time and vary between regions and populations. There have been some studies on the epidemiology of birth defects, such as sex, residence, and maternal age [11–14], and many previous studies have shown that many risk factors may be associated with birth defects, such as environmental factors (e.g., chemical toxicants, infection agents, maternal disease, and exogenous factors), genetic causes (e.g., genetic chromosomal aberrations and dysgeneses ), and socio-economic factors [9, 15]. However, there are also limitations in these studies. First, the epidemiological characteristics and risk factors of birth defects may differ in different regions. Second, some studies were limited in data, such as relatively few cases included or surveys conducted in unrepresentative districts or hospitals. Third, risk factors for birth defects may change over time, so some studies need to be updated. Fourth, there may be interaction (such as synergism and antagonism) for some factors, while few studies have addressed the interaction.

Hospital- and population-based surveillance are the main methods of birth defects surveillance worldwide [3, 16–18]. In China, hospital-based surveillance (mainly including fetuses between 28 weeks of gestation and seven days postpartum in hospitals) is the main method of birth defects surveillance [3], while population-based surveillance is implemented in some areas [19]. Compared to population-based surveillance, hospital-based surveillance has some limitations. First, the large variation in the hospital birth rate in different regions and the relatively short monitoring period of surveillance for hospital-based surveillance [3] may lower the representativeness of the results. Second, some factors are available in population-based surveillance data but not in hospital-based data, such as paternal age and maternal household registration. Third, information on cases of birth defects and fetuses for hospital-based surveillance is collected separately, which does not allow for multivariate regression analysis of risk factors. Nevertheless, most of the above problems can be well addressed with population-based surveillance.

In China, most studies on the epidemiology or prevalence of birth defects are based on hospital-based surveillance data [3, 20–24], while population-based data are rare [25–28]. Several studies were based on population-based surveillance data in Hunan Province, China. E.g., Lili et al. studied the differences in population birth defects in epidemiology analysis between the rural and urban areas [29]; Lili et al. studied the association between ambient air pollution and birth defects [30]; Hong et al. studied the demographic characteristics and environmental risk factors exposure of birth defects in pregnant women [31]. However, to our knowledge, no study on multivariate logistic regression analysis of risk factors for birth defects was done.

Therefore, we conducted a multivariate logistic regression analysis of risk factors for birth defects by using the population-based birth defects surveillance data in Hunan Province, China, 2014–2020, to explore risk factors for birth defects.

## Methods

### Data sources

The data were derived from the Population-based Birth Defects Surveillance System in Hunan Province, China, which is run by the Hunan Provincial Health Commission. In 2008, the Hunan Provincial Health Commission selected Liuyang County and Shifeng District as population-based birth defects surveillance sites, which had undergone a comprehensive evaluation process by experts before the decision. These two places are in the central Hunan Province, with a resident population of about 1.5-2 million and approximately 20,000 live births per year, and the location, demographics, economic conditions, and healthcare facilities genuinely mirror those of the entire province.

The surveillance population included all live births, stillbirths, infant deaths, and legal termination of pregnancy between 28 weeks gestation and 42 days postpartum, whose mothers lived in Liuyang County and Shifeng District between 2014 and 2020. Surveillance data included demographic characteristics such as sex, residence, parents’ age, and other key information. In this study, almost all available demographic characteristics that may be associated with birth defects were chosen for analysis, including sex, residence, number of births, paternal age, maternal age, number of pregnancies, parity, and maternal household registration.

The maternal and child healthcare workers at the community health service centers in urban areas and village doctors in rural areas are responsible for collecting surveillance data. They follow up with the live infants until 42 days after birth. According to the “Maternal and Child Health Monitoring Manual in Hunan Province (2013 Edition)” formulated by the Hunan Provincial Health Commission, diagnostic methods for birth defects included clinical examination, ultrasonography, biochemical examination, chromosomal analysis, genetic testing, autopsy, and other appropriate examination. All birth defects should be diagnosed by medical institutions above the district or county level as soon as possible. Each quarter, county-level surveillance centers will collect the surveillance data and submit it to municipal surveillance centers for review, which then submit it to the provincial surveillance centers (the Hunan Provincial Maternal and Child Health Care Hospital) for review.

Birth defects are coded according to the WHO International Classification of Diseases (Tenth Revision, ICD-10, codes Q00–Q99). The ICD codes of common specific defects are as follows: congenital heart defects (Q20-Q26), congenital metabolic disorders, congenital metabolic disorders (E03, E25.0, E70-E90, D55.0), congenital limb defects (Q69-Q74), congenital ear defects (Q16-Q17), congenital kidney and urinary defects (Q60-Q64), cleft lip and/or palate (Q35-Q37), chromosomal abnormalities (Q90-Q99), congenital digestive system defects (Q38-Q45), congenital nervous system defects (Q00-Q07), and other unclassified defects (Q00–Q99, excluding the codes mentioned above).

### Data quality control

To carry out surveillance, the Hunan Provincial Health Commission formulated the “Maternal and Child Health Monitoring Manual in Hunan Province”, and all levels of government support this work. Data were collected and reported by experienced data collectors. To ensure data consistency and accuracy, all data collectors must be trained and qualified before starting work. To reduce integrity and information error rates of surveillance data, the Hunan Provincial Health Commission asked the technical guidance departments to conduct comprehensive quality control each year. To ensure that all cases of birth defects are accurately diagnosed and classified, all cases are reviewed by provincial doctors.

### Statistical analysis

The prevalence of birth defects (number of birth defects per 1000 infants (lives and deaths)) and its 95% confidence interval (CI) were calculated by the log-binomial method. Chi-square trend tests (*χ*^*2*^_*trend*_) were used to determine trends in the prevalence of birth defects by year. Unadjusted odds ratios (ORs) were calculated to examine the association of each demographic characteristic with birth defects. Multivariate logistic regression analysis (method: Forward, Wald, α = 0.05) and adjusted ORs were used to identify risk factors for birth defects. We used the presence or absence of birth defects (or specific defects) as the dependent variable, and the variables assessed significantly in univariate analysis were entered as independent variables in multivariate logistic regression analysis.

Statistical analyses were performed using SPSS 18.0 (IBM Corp., NY, USA).

## Results

### Prevalence of birth defect in Hunan Province, China, 2014–2020

Our study included 143,118 infants, and 2984 birth defects were identified, with a prevalence of 20.85% (95%CI: 20.10–21.60). From 2014 to 2020, the prevalences of birth defects were 25.74%, 29.29%, 14.73%, 21.76%, 18.45%, 16.03%, and 16.18%, respectively, showing a downward trend (χ^2^_trend_ = 90.66, *P* < 0.01) (Table 1).

### Prevalence of specific defects

Congenital heart defects were the most common specific defects, with a prevalence of 6.39% (95%CI: 5.97–6.80). The prevalences of congenital metabolic disorders, congenital limb defects, congenital ear defects, congenital kidney and urinary defects, cleft lip and/or palate, chromosomal abnormalities, congenital digestive system defects, and congenital nervous system defects were 4.29% (95%CI: 3.95–4.63), 4.20% (95%CI: 3.86–4.54), 1.51% (95%CI: 1.31–1.71), 1.10% (95%CI: 0.93–1.27), 0.75% (95%CI: 0.61–0.90), 0.49% (95%CI: 0.37–0.60), 0.44% (95%CI: 0.33–0.55), and 0.43% (95%CI: 0.32–0.53), respectively. Table 2 shows the details of the prevalence of specific defects (Table 2).

### Demographic characteristics of birth defects

Birth defects were more common in males than females (24.59% vs. 16.66%, OR = 1.49, 95%CI: 1.38–1.60), in multiple births than singleton (29.21% vs. 20.64%, OR = 1.43, 95%CI: 1.17–1.74), in maternal non-local household registration than local household registration (58.52% vs. 20.43%, OR = 2.98, 95%CI: 2.41–3.69), while were less common in parity is 2 than 1 (19.96% vs. 21.90%, OR = 0.91, 95%CI: 0.84–0.98). Compared to paternal age 25–29, birth defects were more common in < 20 (44.94% vs. 22.83%, OR = 2.01, 95%CI: 1.13–3.60) or 20–24 (33.92% vs. 22.83%, OR = 1.50, 95%CI: 1.30–1.73), while less common in 30–34 (17.46% vs. 22.83%, OR = 0.76, 95%CI: 0.70–0.83) or > = 35 (20.07% vs. 22.83%, OR = 0.88, 95%CI: 0.80–0.96). Compared to maternal age 25–29, birth defects were more common in < 20 (28.35% vs. 20.19%, OR = 1.42, 95%CI: 1.10–1.82) or > = 35 (24.56% vs. 20.19%, OR = 1.22, 95%CI: 1.09–1.37). However, there were no significant difference in prevalence between urban and rural areas (20.62% vs. 20.97%), maternal age 20–24 and 25–29 (21.90% vs. 20.19%), maternal age 25–29 and 30–34 (20.19% vs. 19.30%), number of pregnancies is 1, 2, 3, and > = 4 (21.49% vs. 19.97% vs. 21.24% vs. 21.62%), or parity is 1 and > = 3 (21.90% vs. 21.41%). Table 3 shows the details of demographic characteristics (Table 3).

### Multivariate logistic regression analysis of risk factors for birth defects

In the univariate analysis, all demographic characteristics were associated with birth defects except for residence and number of pregnancies (Table 3). However, some previous studies have shown that residence and number of pregnancies were associated with birth defects (or some specific defects). In addition, we believed that confounding associations could be effectively corrected by multivariate logistic regression analysis. Therefore, all variables in Table 3 were entered as independent variables in multivariate logistic regression analysis. The variables are coded as shown in Table 4. As a result, seven variables (except for parity) were associated with birth defects (or specific defects). (Table 5).

Overall, five variables (sex, number of births, paternal age, maternal age, and maternal household registration) were associated with overall birth defects. The risk factors included males (OR = 1.49, 95%CI: 1.39–1.61), multiple births (OR = 1.44, 95%CI: 1.18–1.76), paternal age < 20 (OR = 2.20, 95%CI: 1.19–4.09) or 20–24 (OR = 1.66, 95%CI: 1.42–1.94), maternal age 30–34 (OR = 1.16, 95%CI: 1.04–1.29) or > = 35 (OR = 1.56, 95%CI: 1.33–1.81), and maternal non-local household registration (OR = 2.96, 95%CI: 2.39–3.67).

Some factors were associated with the specific defects. As shown in Table 5, four variables (number of births, paternal age, maternal age, and maternal household registration) were associated with congenital heart defects, five variables (sex, residence, paternal age, maternal age, and maternal household registration) were associated with congenital metabolic disorders, four variables (sex, paternal age, maternal age, and maternal household registration) were associated with congenital limb defects, four variables (residence, paternal age, maternal age, and maternal household registration) were associated with congenital ear defects, two variables (sex and number of births) were associated with congenital kidney and urinary defects, two variables (number of births and maternal age) were associated with cleft lip and/or palate, one variable (number of pregnancies) was associated with chromosomal abnormalities, two variables (paternal age and number of pregnancies) were associated with congenital nervous system defects, and no variable was associated with congenital digestive system defects.

Males were risk factors for congenital metabolic disorders (OR = 3.86, 95%CI: 3.15–4.72), congenital limb defects (OR = 1.34, 95%CI: 1.14–1.58), and congenital kidney and urinary defects (OR = 2.35, 95%CI: 1.65–3.34). Rural areas were risk factors for congenital metabolic disorders (OR = 1.21, 95%CI: 1.01–1.44). Multiple births were risk factors for congenital heart defects (OR = 2.09, 95%CI: 1.55–2.82), congenital kidney and urinary defects (OR = 2.14, 95%CI: 1.05–4.37), and cleft lip and/or palate (OR = 2.85, 95%CI: 1.32–6.15). Paternal age < 20 was the risk factor for congenital limb defects (OR = 3.27, 95%CI: 1.10–9.71), 20–24 was the risk factor for congenital heart defects (OR = 1.64, 95%CI: 1.24–2.17), congenital metabolic disorders (OR = 1.56, 95%CI: 1.11–2.21), congenital limb defects (OR = 1.61, 95%CI: 1.14–2.29), and congenital ear defects (OR = 2.13, 95%CI: 1.17–3.89). Maternal age < 20 was the risk factor for cleft lip and/or palate (OR = 3.14, 95%CI: 1.24–7.95), 30–34 was the risk factor for congenital limb defects (OR = 1.37, 95%CI: 1.09–1.73), >=35 was the risk factor for congenital heart defects (OR = 1.51, 95%CI: 1.14–1.99), congenital limb defects (OR = 1.98, 95%CI: 1.41–2.78), and congenital ear defects (OR = 1.82, 95%CI: 1.06–3.10). Number of pregnancies = 2 was the risk factor for congenital nervous system defects (OR = 2.27, 95%CI: 1.19–4.32), >=4 was the risk factor for chromosomal abnormalities (OR = 2.03, 95%CI: 1.06–3.88) and congenital nervous system defects (OR = 3.03, 95%CI: 1.23–7.47). Maternal non-local household registration was the risk factor for congenital heart defects (OR = 3.57, 95%CI: 2.54–5.03), congenital metabolic disorders (OR = 1.89, 95%CI: 1.06–3.37), congenital limb defects (OR = 2.94, 95%CI: 1.86–4.66), and congenital ear defects (OR = 3.26, 95%CI: 1.60–6.65) (Table 5).

## Discussion

Overall, several risk factors are associated with birth defects, and the risk factors vary dramatically across specific defects. Our study is the most recent systematic study on risk factors for birth defects by using multivariate logistic regression analysis from population-based surveillance data in Hunan Province, China. Our discovery makes a significant original contribution to the field.

There were several meaningful findings in this study. First, most previous studies have shown a higher prevalence of birth defects in urban areas than in rural areas [22, 32, 33], including some specific defects, such as congenital heart defects [20] and congenital limb defects (polydactyly and syndactyly) [34, 35], ear defects (anotia and microtia) [36]. However, no statistical difference was shown in this study. It may be mainly related to the surveillance methods. Most previous studies were based on hospital-based surveillance, so higher diagnosis and reporting rates in urban areas may lead to a higher prevalence of birth defects [33]. In comparison, this study was based on population-based surveillance, with relatively long surveillance periods, avoiding these problems. In addition, in this study, rural areas were risk factors for congenital metabolic disorders, which is consistent with previous studies [37]. It may be mainly related to low health education and understanding relevant preventive measures for congenital metabolic disorders [37].

Second, low paternal age was a risk factor for birth defects in this study. While some previous studies have addressed the relationship between maternal age and birth defects [15, 38], fewer studies have addressed the relationship between paternal age and birth defects. Moreover, we have analyzed the relationship between a broad range of specific defects and paternal age, and some meaningful findings were obtained, which makes a significant original contribution to risk factors for birth defects.

Third, in this study, maternal non-local household registration was a risk factor for overall birth defects and the main specific defects. Similar to paternal age, maternal household registration as an indicator has rarely been addressed in previous studies. Yu-Jung et al. found that the prevalence of birth defects in newborns of immigrant mothers was lower than that of native-born mothers in Taiwan [39]. In contrast, Anne-Marie et al. found a higher risk of congenital anomalies in migrants in Europe [40]. We infer that maternal household registration is primarily the reflection of socio-economic conditions. In general, people with non-local household registration are mainly migrant workers, who may have poorer economic, medical, living, and working conditions than those with local household registration, which may contribute to birth defects. Although the proportion of non-local household residents in our study was relatively low, the proportion of non-local household residents was high in some areas of China. Our study is informative for other regions.

Fourth, unlike the overall birth defects, sex or number of births were not associated with some predominant specific defects. E.g., there was no difference in sex for congenital heart defects, which was consistent with previous studies [41]; no difference in sex for ear defects, which seems inconsistent with previous studies [42, 43]; multiple births were not a risk factor for congenital metabolic disorders, congenital limb defects, and congenital ear defects, which seems inconsistent with previous studies [44] or rarely addressed. Those differences may also be related to the methods of surveillance and analysis. This study makes a significant original contribution to risk factors for specific defects.

Fifth, unlike the overall birth defects, some specific defects were associated with only a few factors. E.g., risk factors for congenital kidney and urinary defects included only males and multiple births, which was rarely addressed [45, 46]. Brouwers et al. found multiple births were a risk factor for hypospadias [47], similar to this study. Risk factors for cleft lip and/or palate included only multiple births and low maternal age (< 20), which was consistent with previous studies [48, 49]. However, some factors (such as advanced paternal age) were associated with cleft lip and/or palate in some studies [49], but not in this study. High number of pregnancies ( > = 4 times) was the only risk factor for chromosomal abnormalities. Most previous studies have concluded that the risks of chromosomal abnormalities increase with advancing maternal age [50–52]. In this study, maternal age was also included in the analysis. However, the multivariate logistic regression analysis showed that advanced maternal age was not a risk factor for chromosomal abnormalities. We believe that the high number of pregnancies may be mainly related to spontaneous miscarriage, and many were recurrent miscarriages, which may be associated with chromosomal abnormalities [53–55]. As the mechanism by which advanced maternal age leads to chromosomal abnormalities is unclear [50], maternal age cannot be ruled out as a confounding factor for chromosomal abnormalities, while the high number of pregnancies is the exact risk factor for chromosomal abnormalities. Our findings provide clues for mechanistic studies. Risk factors for congenital nervous system defects included only low paternal age and number of pregnancies (2 or > = 4 times), which was rarely addressed [56, 57]. Some studies found that males and low or advanced maternal age were risk factors for congenital nervous system defects [11, 58], while not in this study. No factor was associated with congenital digestive system defects, inconsistent with previous studies [59, 60]. The few available epidemiological studies demonstrated that some factors may increase the risk of congenital digestive system defects, such as obesity and diabetes [60, 61].

**Final**ly, the above findings suggest that birth defects may result from gene-environment interactions. The differences between our findings and those of previous studies suggest differences in the factors that lead to birth defects, which requires in-depth research. Our study provides clues for further mechanism studies.

Some things could be improved in our study. First, although some meaningful results were found, the association between risk factors and birth defects only showed a correlation but not a cause-and-effect link. Further studies should examine the mechanism. Second, our study did not include birth defects before 28 weeks of gestation. A considerable proportion of birth defects are diagnosed and terminated before 28 weeks of gestation (such as Down syndrome), which significantly impacts the prevalence and risk factors of birth defects. Third, some factors were not included in our study, such as parental weight, illness during pregnancy, and medication use during pregnancy, which may be potential risk factors for birth defects. Fourth, there are some multiple birth defects in this study, some risk factors may be associated with multiple defects, and studies on multiple birth defects may be more sensitive to finding risk factors. However, the mechanism and analysis may be complicated and need in-depth research in the future. Fifth, to our knowledge, no study on risk factors of birth defects based on national data, while the representativeness of this study population for the whole of China is questionable.

## Conclusion

In summary, several risk factors were associated with birth defects (including a broad range of specific defects). One risk factor may be associated with several defects, and one defect may be associated with several risk factors. Future studies should examine the mechanisms. Our findings have significant public health implications as some factors are modifiable or avoidable, such as promoting childbirths at the appropriate age, improving the medical and socio-economic conditions of non-local household registration residents, and devoting more resources to some specific defects in high-risk groups, which may help reducing birth defects in China.

**Table 1:** Prevalence of birth defect in Hunan Province, China, 2014–2020

Year	Infants (n)	Birth defects (n)	Prevalence (%, 95%CI)
2014	22,299	574	25.74(23.64–27.85)
2015	23,284	682	29.29(27.09–31.49)
2016	22,943	338	14.73(13.16–16.30)
2017	24,863	541	21.76(19.93–23.59)
2018	20,654	381	18.45(16.59–20.30)
2019	16,035	257	16.03(14.07–17.99)
2020	13,040	211	16.18(14.00-18.36)
Total	143,118	2984	20.85(20.10–21.60)

*Abbreviations* CI: confidence intervals

**Table 2:** Prevalence of specific defects

Types	Infants (n)	Birth defects (n)	Prevalence (%, 95%CI)
Congenital heart defects	143,118	914	6.39(5.97–6.80)
Congenital metabolic disorders	143,118	614	4.29(3.95–4.63)
Congenital limb defects	143,118	601	4.20(3.86–4.54)
Polydactyly	143,118	330	2.31(2.06–2.55)
Clubfoot	143,118	125	0.87(0.72–1.03)
Syndactyly	143,118	78	0.55(0.42–0.67)
Limb reduction	143,118	37	0.26(0.18–0.36)
Other limb defects	143,118	43	0.30(0.22–0.40)
Congenital ear defects	143,118	216	1.51(1.31–1.71)
Anotia/microtia	143,118	59	0.41(0.31–0.52)
Other external ear defects	143,118	164	1.15(0.97–1.32)
Congenital kidney and urinary defects	143,118	157	1.10(0.93–1.27)
Kidney defects	143,118	88	0.61(0.49–0.74)
Hypospadias	143,118	68	0.48(0.36–0.59)
Other kidney and urinary system defects	143,118	1	0.01(0.00-0.04)
Cleft lip and/or palate	143,118	108	0.75(0.61–0.90)
Cleft lip with palate	143,118	39	0.27(0.19–0.37)
Cleft palate	143,118	49	0.34(0.25–0.45)
Cleft lip	143,118	20	0.14(0.09–0.22)
Chromosomal abnormalities	143,118	70	0.49(0.37–0.60)
Down syndrome	143,118	28	0.20(0.13–0.28)
Other chromosomal abnormalities (aneuploid chromosomes or large chromosome deletions)	143,118	42	0.29(0.21–0.40)
Congenital digestive system defects	143,118	63	0.44(0.33–0.55)
Anal atresia	143,118	31	0.22(0.15–0.31)
Esophageal atresia	143,118	6	0.04(0.02–0.09)
Other digestive system defects	143,118	26	0.18(0.12–0.27)
Congenital nervous system defects	143,118	61	0.43(0.32–0.53)
Hydrocephalus	143,118	31	0.22(0.15–0.31)
Spina bifida	143,118	9	0.06(0.03–0.12)
Other nervous system defects	143,118	23	0.16(0.10–0.24)
Other unclassified defects	143,118	301	2.10(1.87–2.34)

*Abbreviations* CI: confidence intervals

*Note* A total of 130 infants (4.36%) were diagnosed with multiple birth defects. In the table, if the sum of the number of subtypes is higher than the total number, it suggests that it contains multiple defects

**Table 3:** Prevalence of birth defects by demographic characteristics (univariate analysis)

Variables	Infants		Birth defects		Unadjusted OR (95%CI)
	n	Proportion in total (%)	n	Prevalence (%, 95%CI)	
Sex					
Female	67,809	47.38	1130	16.66(15.69–17.64)	Reference
Male	75,305	52.62	1852	24.59(23.47–25.71)	1.49(1.38–1.60)
Unknown	4	< 0.01	2	-	-
Residence					
Urban	47,777	33.38	985	20.62(19.33–21.90)	Reference
Rural	95,341	66.62	1999	20.97(20.05–21.89)	1.02(0.94–1.10)
Number of births					
Singleton	139,557	97.51	2880	20.64(19.88–21.39)	Reference
Multiple births	3561	2.49	104	29.21(23.59–34.82)	1.43(1.17–1.74)
Paternal age (years old)					
25–29	51,332	35.87	1172	22.83(21.52–24.14)	Reference
< 20	267	0.19	12	44.94(19.51–70.37)	2.01(1.13–3.60)
20–24	6928	4.84	235	33.92(29.58–38.26)	1.50(1.30–1.73)
30–34	50,809	35.50	887	17.46(16.31–18.61)	0.76(0.70–0.83)
>=35	33,782	23.60	678	20.07(18.56–21.58)	0.88(0.80–0.96)
Maternal age (years old)					
25–29	62,406	43.60	1260	20.19(19.08–21.31)	Reference
< 20	2328	1.63	66	28.35(21.51–35.19)	1.42(1.10–1.82)
20–24	24,523	17.13	537	21.90(20.05–23.75)	1.09(0.98–1.20)
30–34	38,391	26.82	741	19.30(17.91–20.69)	0.96(0.87–1.05)
>=35	15,470	10.81	380	24.56(22.09–27.03)	1.22(1.09–1.37)
Number of pregnancies (times)					
1	48,541	33.92	1043	21.49(20.18–22.79)	Reference
2	57,947	40.49	1157	19.97(18.82–21.12)	0.93(0.85–1.01)
3	20,995	14.67	446	21.24(19.27–23.21)	0.99(0.88–1.11)
>=4	15,635	10.92	338	21.62(19.31–23.92)	1.01(0.89–1.14)
Parity (times)					
1	60,271	42.11	1320	21.90(20.72–23.08)	Reference
2	75,934	53.06	1516	19.96(18.96–20.97)	0.91(0.84–0.98)
>=3	6913	4.83	148	21.41(17.96–24.86)	0.98(0.82–1.16)
Maternal household registration					
Local	141,546	98.90	2892	20.43(19.69–21.18)	Reference
Non-local	1572	1.10	92	58.52(46.57–70.48)	2.98(2.41–3.69)
Total	143,118	100.00	2984	20.85(20.10–21.60)	

*Abbreviations* CI: confidence intervals; OR: odds ratio

**Table 4:** Methods of coding for variables in the multivariate logistic regression analysis

Variables	Types	Codes, values
Overall birth defects or specific defects	Dependent variable	0 = “No” (Reference) 1 = “Yes”
Sex	Independent variable	1 = “Females” (Reference) 2 = “Males” 3 = “Unknown”
Residence	Independent variable	1 = “Urban” (Reference) 2 = “Rural”
Number of births	Independent variable	1 = “Singleton” (Reference) 2 = “Multiple births”
Paternal age	Independent variable	1 = “25–29 years old” (Reference) 2 = “<20 years old” 3 = “20–24 years old” 4 = “30–34 years old” 5 = “>=35 years old”
Maternal age	Independent variable	1 = “25–29 years old” (Reference) 2 = “<20 years old” 3 = “20–24 years old” 4 = “30–34 years old” 5 = “>=35 years old”
Number of pregnancies	Independent variable	1 = “1 time” (Reference) 2 = “2 times” 3 = “3 times” 4 = “>=4 times”
Parity	Independent variable	1 = “1 time” (Reference) 2 = “2 times” 3 = “>=3 times”
Maternal household registration	Independent variable	1 = “Local” (Reference) 3 = “Non-local”

**Table 5:** Multivariate logistic regression analysis of risk factors for birth defects (Forward, Wald, α = 0.05)

Variables	Adjusted OR (95%CI)									
	Overall birth defects	Congenital heart defects	Congenital metabolic disorders	Congenital limb defects	Congenital ear defects	Congenital kidney and urinary defects	Cleft lip and/or palate	Chromosomal abnormalities	Congenital digestive system defects	Congenital nervous system defects
Sex		Not entered			Not entered		Not entered	Not entered	Not entered	Not entered
Females	Reference		Reference	Reference		Reference				
Males	1.49(1.39–1.61)		3.86(3.15–4.72)	1.34(1.14–1.58)		2.35(1.65–3.34)				
Unknown	50.82(7.01-368.28)		Counter overflow	Counter overflow		1621.08(223.15-11776.22)				
Residence	Not entered	Not entered		Not entered		Not entered	Not entered	Not entered	Not entered	Not entered
Urban			Reference		Reference					
Rural			1.21(1.01–1.44)		0.76(0.58-1.00)					
Number of births			Not entered	Not entered	Not entered			Not entered	Not entered	Not entered
Singleton	Reference	Reference				Reference	Reference			
Multiple births	1.44(1.18–1.76)	2.09(1.55–2.82)				2.14(1.05–4.37)	2.85(1.32–6.15)			
Paternal age (years old)						Not entered	Not entered	Not entered	Not entered	
25–29	Reference	Reference	Reference	Reference	Reference					Reference
< 20	2.20(1.19–4.09)	2.34(0.70–7.85)	Counter overflow	3.27(1.10–9.71)	4.45(0.52–38.22)					Counter overflow
20–24	1.66(1.42–1.94)	1.64(1.24–2.17)	1.56(1.11–2.21)	1.61(1.14–2.29)	2.13(1.17–3.89)					2.14(0.93–4.92)
30–34	0.69(0.63–0.77)	0.73(0.61–0.87)	0.63(0.50–0.78)	0.65(0.52–0.80)	0.54(0.37–0.78)					0.57(0.31–1.03)
>=35	0.66(0.57–0.75)	0.65(0.51–0.83)	0.72(0.54–0.96)	0.50(0.37–0.67)	0.62(0.39-1.00)					0.27(0.12–0.64)
Maternal age (years old)						Not entered		Not entered	Not entered	Not entered
25–29	Reference	Reference	Reference	Reference	Reference		Reference			
< 20	0.89(0.67–1.18)	0.62(0.35–1.12)	1.21(0.72–2.06)	1.00(0.56–1.78)	0.42(0.11–1.52)		3.14(1.24–7.95)			
20–24	0.86(0.77–0.96)	0.94(0.77–1.14)	0.74(0.58–0.96)	0.80(0.62–1.03)	0.57(0.35–0.91)		1.44(0.87–2.37)			
30–34	1.16(1.04–1.29)	1.20(1.00-1.45)	1.02(0.81–1.30)	1.37(1.09–1.73)	1.39(0.95–2.04)		0.74(0.44–1.26)			
>=35	1.56(1.33–1.81)	1.51(1.14–1.99)	1.38(0.99–1.92)	1.98(1.41–2.78)	1.82(1.06–3.10)		1.48(0.84–2.64)			
Number of pregnancies (times)	Not entered	Not entered	Not entered	Not entered	Not entered	Not entered	Not entered		Not entered	
1								Reference		Reference
2								0.76(0.42–1.38)		2.27(1.19–4.32)
3								1.11(0.54–2.27)		2.13(0.91–4.99)
>=4								2.03(1.06–3.88)		3.03(1.23–7.47)
Parity (times)	Not entered	Not entered	Not entered	Not entered	Not entered	Not entered	Not entered	Not entered	Not entered	Not entered
1										
2										
>=3										
Maternal household registration						Not entered	Not entered	Not entered	Not entered	Not entered
Local	Reference	Reference	Reference	Reference	Reference					
Non-local	2.96(2.39–3.67)	3.57(2.54–5.03)	1.89(1.06–3.37)	2.94(1.86–4.66)	3.26(1.60–6.65)					

*Abbreviations* OR: odds ratio; CI: confidence intervals

## Data Availability

The datasets used and/or analyzed during the current study are available from the corresponding author on reasonable request.
